# Different time patterns of the presence of red-eared slider influence the ontogeny dynamics of common frog tadpoles

**DOI:** 10.1038/s41598-022-11561-6

**Published:** 2022-05-12

**Authors:** M. Vodrážková, I. Šetlíková, J. Navrátil, M. Berec

**Affiliations:** Faculty of Agriculture and Technology, University of South Bohemia in České Budějovice, Studentská 1668, České Budějovice, Czech Republic

**Keywords:** Ecology, Zoology, Ecology, Environmental sciences, Hydrology

## Abstract

The coexistence of species in a given community depends on the set of species involved and the timing of their interactions. Many native communities are increasingly forced to face both direct and indirect pressures from new alien predators, which, in extreme cases, can lead to the extinction of prey populations. In this study, we examine the dynamics of the ontogeny of common frog (*Rana temporaria*) tadpoles under different time patterns of an alien predator—the red-eared slider (*Trachemys scripta elegans*) presence. We found that the tadpoles had a longer larval period and were smaller in size at metamorphosis and lower in body mass when the predator was present in early development than when the tadpoles developed without a predator. The early presence of a predator conspicuously reduced the growth increments of the tadpoles at early development. After the removal of the predator, growth accelerated above the level measured under the conditions of both the late predator and no predator. However, these growth rates did not exceed the growth rates of equally sized tadpoles in the other treatments and therefore were not sufficient to compensate for the growth slowdown in the first part of development. The presence of a predator in late tadpole development influenced neither the time to metamorphosis nor size/body mass at metamorphosis. In conclusion, the predator had the effect on metamorphosis traits only if it was present in the early development of tadpoles.

## Introduction

The coexistence of species in a given community depends on the set of species involved^1,2^. Any change in species composition can have consequences on interacting species, both within and among trophic levels^3–5^, the result of which depends on the timing of presence/activity of individual species^6–9^. Globally, many native communities are currently increasingly forced to face new alien predators^10^, which, in extreme cases, can lead to the extinction of whole prey populations^11,12^. In addition to these predators causing direct mortality, current theoretical and empirical studies of trophic cascades suggest that the indirect effect of the "landscape of fear" created by predators may be more important than direct killing^13–16^.

Amphibians are a particularly good model for studying morphological, physiological, and behavioural responses to changes in community composition^17^. These responses can, inter alia, consist of changes in body morphology^18,19^, behaviour^20^, or timing of life-history switch points, such as hatching^21–23^ or metamorphosis^24–26^. Specifically, predation during early life-history stages, when organisms are particularly susceptible to both biotic and abiotic factors, is likely to have pervasive effects on community development^27^. The fitness consequences of phenological shifts that appear early in the ontogeny of a species can also be fundamentally different from the effects observed at later ontogenetic stages. For example, the relatively early seasonal appearance (or accelerated developmental rate) of a prey species may decrease its predation risk early in life, but increase the negative effects of competition with its former predator later in life^28^.

Tadpoles of various frog species respond differently to novel predators. Some species innately detect and elicit adaptive responses to stimuli of novel predators in the absence of a common evolutionary history^29–31^. In others, no such responses have been documented^32,33^. One of the 100 worst world invaders^34^ is a red-eared slider (*Trachemys scripta elegans*), which is also among the most widespread animal species outside its native range^35,36^. Berec, et al. ^37^ and Vodrážková, et al.^38^ recently found a response in the behaviour and growth of common frog (*Rana temporaria*) tadpoles to the presence of red-eared sliders in the form of reduced movement activity associated with more zigzagged movement trajectories, smaller size at metamorphosis, and shorter time to metamorphosis. In these studies, the response of the common frog tadpoles was examined under the continuous presence of a red-eared slider throughout larval development. However, such a situation may not correspond to reality, as turtles frequently change their place of occurrence in a habitat during a period of activity^39^; therefore, at least in areas where the red-eared slider does not naturally reproduce and thus its density is relatively low, different populations may encounter red-eared sliders at different parts of development and for different time intervals.

In this study, we examine the dynamics of the ontogeny of common frog tadpoles under different time patterns of red-eared slider presence. Because the presence of red-eared sliders reduces the activities of tadpoles and consequently their growth rates^37,38^, we expect that the presence of turtles in any part of larval ontogenesis will lead to a growth slowdown. As the level of this slowdown decreases with increasing size as an effect of better movement abilities in terms of faster escape reaction to predator^40–42^, we expect that the presence of red-eared sliders in the early stages of ontogeny will have a more pronounced effect than their presence in the later stages. In the early red-eared slider presence, this could lead to a smaller size and/or weight of tadpoles at metamorphosis with the same time to metamorphosis as in tadpoles with the late red-eared slider presence^43^ or to the same size and weight of tadpoles at metamorphosis with a longer time to metamorphosis than the tadpoles with the late red-eared slider presence^44^. Alternatively, if it is more advantageous for tadpoles to leave the hazardous environment with tadpoles as soon as possible, we might expect a reduction in the time to metamorphosis together with a smaller total size.

## Materials and methods

Common frog tadpoles originated from six clutches that were collected in pools near Holubov, South Bohemia, Czech Republic (48.9078633°N, 14.3485608°E) on 5 April 2020. Clutches were placed in a 220 L glass tank with tap water in a temperature-controlled laboratory. Each glass tank was equipped with a pump filter that was cleaned twice a week along with removing debris from the bottom of the tanks, and the tanks were replenished with an equal volume of the tap water (up to 5% of glass tank volume). The acclimatization temperature was gradually raised from 16 to 21 °C for fourteen days. The tadpoles were fed ad libitum with TetraMin aquarium flakes for ornamental fish. The light source was fluorescent tubes (2 × 36 W) with a light regime of 12 h/12 h.

Six glass tanks that were each 220 L (size: 100 × 55 × 40 cm) with an 8 cm water depth were used for the experiment. A Claro 300 filter pump (300 L h^−1^) was installed in each glass tank. Two adult red-eared sliders (carapax length: 21 cm and 18 cm) obtained from Hluboká nad Vltavou zoo were used as the predators. Turtles were placed in two of the glass tanks before the experiment and were fed three times a week with ReptoMin Tetra turtle food consisting of *Gammarus*. To prevent physical, but not chemical, contact between a turtle and tadpoles, a glass barrier was placed inside each glass tank three days before the start of the experiment with a 6 cm gap at both ends so that water could flow freely throughout the tank. On the other side of the barriers, 21 individual perforated opaque boxes (8 × 8 cm) with holes 2 mm in diameter were glued to the bottoms of the glass tanks (7 rows with 3 boxes).

Tadpole development was observed in three treatments, each with two independent repetitions (glass tanks). Based on a pilot experiment, the development time at a given temperature was determined to be approximately six weeks. In the first treatment, tadpoles were present in the tanks with red-eared sliders for the first three weeks of development (predator early—PE). In the second treatment, tadpoles developed with turtles from the start of the 4th week until the completion of metamorphosis (predator late—PL). At the time of the switch of predator presence, the tadpoles of the PE treatment were exchanged with those of the PL treatment and vice versa. This transfer of tadpoles took place during the regular 7-day measurement period (see below), so handling was the same in all treatments, although the tadpoles were returned to the same glass tank in the control. Water maintenance in the glass tanks was carried out at regular intervals, that is, three days before the replacement of tadpoles. In the control, the tadpoles developed without a slider all the time. Neither the eggs nor their parents came into contact with a red-eared slider at the collection locality.

At the beginning of the experiment, similarly sized tadpoles (18.9 ± 1.27 mm) at stages 26 and 27 according to Gosner^45^ were stocked individually in each box. During the experiment, the same feeding and light regime as that in the acclimation phase was used. The water temperature was 21 ± 1.7 °C (mean ± S.D.). During the experiment, the tadpoles were photographed under a stereomicroscope (Olympus SZX 7) and measured (to the nearest 0.5 mm) using QuickPHOTO MICRO 3.2 software every seven days. After reaching metamorphosis (Gosner phase 46), the time to metamorphosis was recorded in days, the size of every froglet was measured to the nearest 0.5 mm, and after gently blotting them dry on absorbent paper, each froglet was weighed to the nearest 0.01 g.

To compare the growth trajectories of tadpoles among treatments, the growth rate at a particular measurement (time interval) and the growth rate related to a particular total size (body + tail length) were used. The growth was expressed as a percentage increase in size between the two subsequent measurements. Negative growth increment values, indicating the onset of metamorphosis, were not used. Individual linear growth curves (i.e., the relationship between growth increments and total size) were calculated. To determine the ability to compensate for the reduced growth rate in the presence of a predator in the PE treatment, the slopes and intercepts of individual growth curves (percentage of growth increment versus size) were compared among the PE treatment, the PL treatment and the control. To eliminate the effect of absolute total size (due to the upper size limit, i.e., larger tadpoles have less potential growth capacity), we compared growth curves only in the tadpole size range detected in the turtle-free period (i.e., from the 3rd time interval) in the PE treatment with the tadpole growth rate of the same size interval in the PL treatment and the control.

The normality of the residuals and homogeneity of the variances were checked using the Shapiro–Wilk test and the Bartlett test, respectively. Differences in time to metamorphosis among the treatments were tested by survival analysis for comparison of multiple groups. To do this, a score was assigned to each survival time using Mantel’s procedure and next a Chi-square value was computed based on the sums (for each group) of this score. Differences in final size and body mass at metamorphosis, the growth increments at particular time intervals of development, and the slopes and intercepts of the individual growth lines among the treatments were tested using repeated measures ANOVA (as tadpoles in one glass tank could not be handled as completely independent one from the another) with predator presence/absence as three-level categorical factor followed by post-hoc comparisons using Tukey HSD method. All data analyses were performed using Survival Analysis and General Linear Models in Tibco Statistica 13.

All methods were carried out in accordance with relevant guidelines and regulations. All experimental protocols were approved by the Czech Ministry of Agriculture, Department of Animal Welfare according to article No. 15, Section 2 of the act registered under number MZP/2019/630/437. Appropriate permission (No. 2/2020) was taken from the zoo authorities for obtaining the turtles. We adhered the recommendations in the ARRIVE guidelines.

## Results

### Metamorphosis time and size

**Figure 1:**
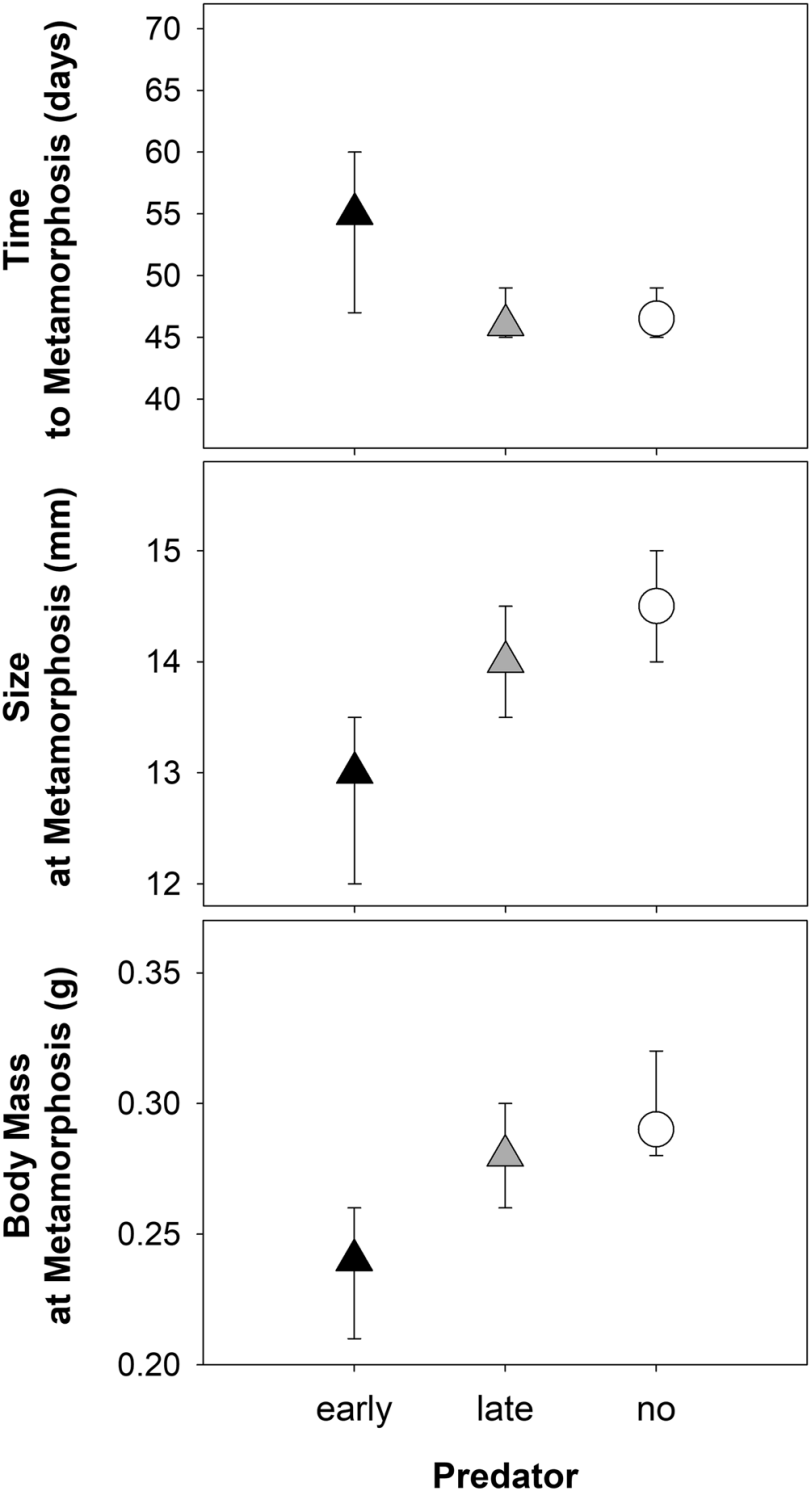
Time to metamorphosis and size and body mass at metamorphosis (median and interquartile range) in the treatments.

Time to metamorphosis (Chi-square = 23.5, df = 2, *p* < 0.001), size (F(2,3) = 25.7, *p* = 0.013) and body mass (F(2,3) = 10.1, *p* = 0.047) at metamorphosis did significantly differ between treatments. Compared to the other treatments, in the PE treatment, tadpoles achieved metamorphosis later and were smaller with respect to their size and body mass (Fig. 1). In contrast, the presence of a predator in the later phase of development had no effect on all factors measured (Fig. 1). Specifically, the tadpoles without red-eared sliders metamorphosed in 46.5 days (median). In comparison to the control, the early presence of a predator delayed metamorphosis by 8.5 days. In the PL treatment, the tadpoles metamorphosed in 46 days. Size and body mass at metamorphosis were the lowest (medians 13.0 mm and 0.24 g) in the PE treatment. Tadpoles metamorphosed at similar size (14.0 mm and 14.5 mm in the PL treatment and the control, respectively) and body mass (0.28 g and 0.29 g in the PL treatment and the control, respectively) in the other two treatments (Fig. 1).

**Figure 2:**
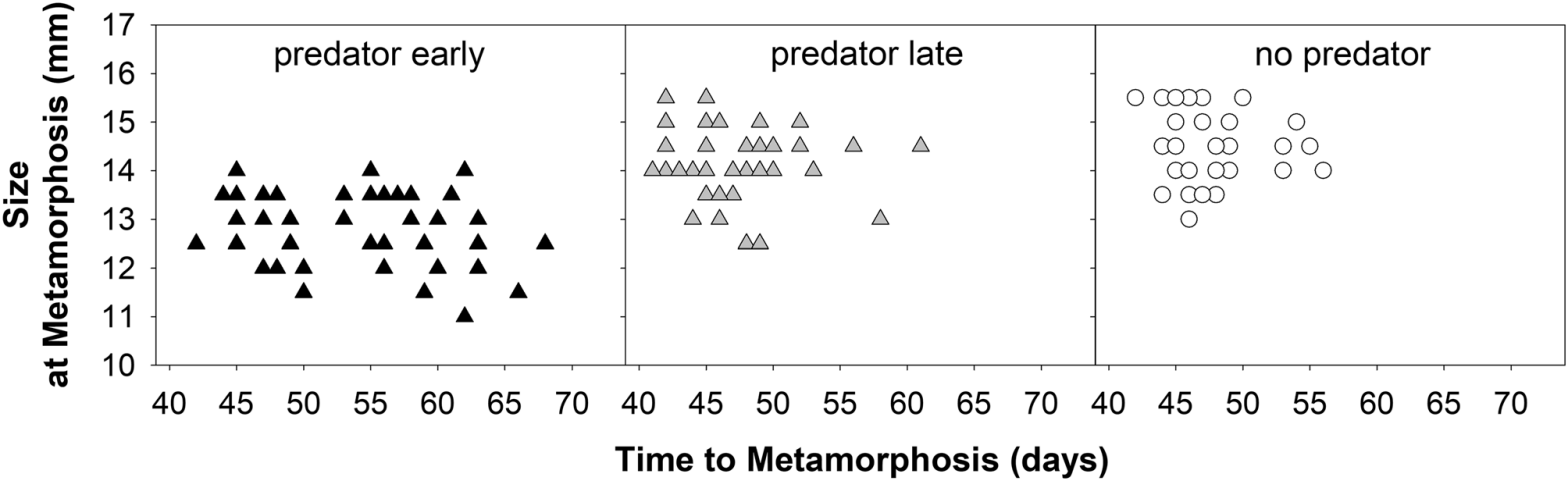
Size at metamorphosis and time to metamorphosis of individual tadpoles in the treatments. Note that some dots overlap.

Furthermore, the presence of a predator increased the interquartile range in the time to metamorphosis of the tadpoles in the PE treatment to 13 days in comparison to 4 days in both the PL treatment and the control, while the interquartile range in size at metamorphosis was comparable among all treatments (Figs. 1 and 2). The first individuals metamorphosed on the 41st day in the PE treatment and on the 42nd day in the PL treatment and the control. The last individual metamorphosed on the 68th day, 61st day and 56th day in the PE treatment, the PL treatment and the control, respectively (Fig. 2).

### Growth of tadpoles

**Figure 3:**
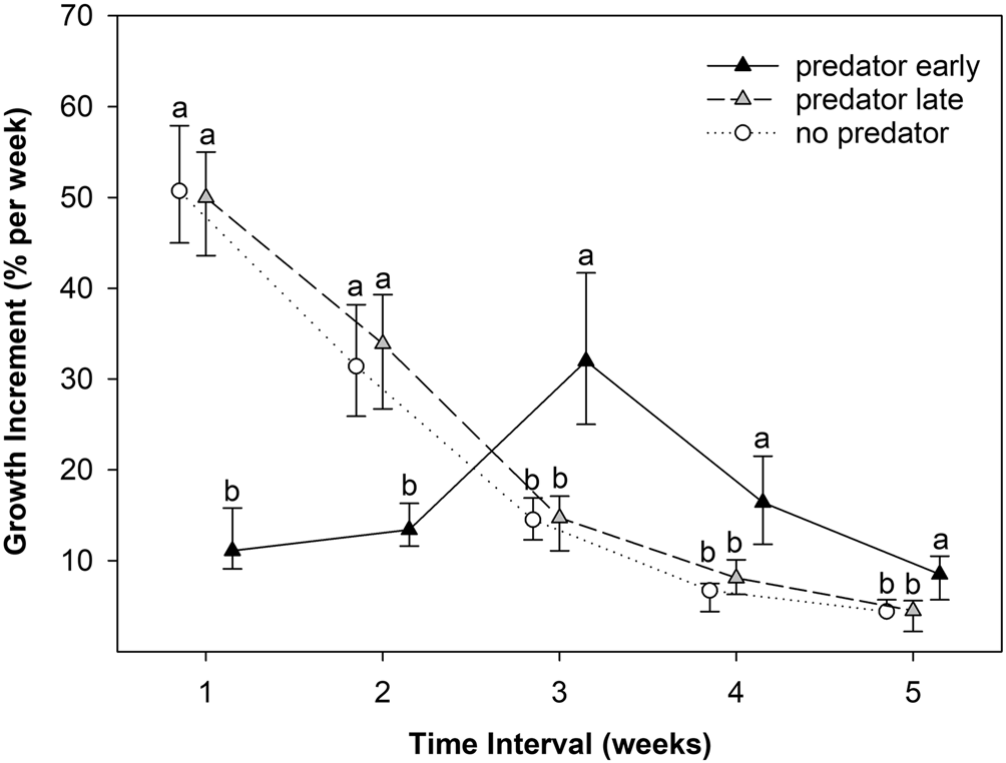
Median and interquartile range of growth increment in particular time intervals in the treatments. Different letters indicate a significant difference.

**Figure 4:**
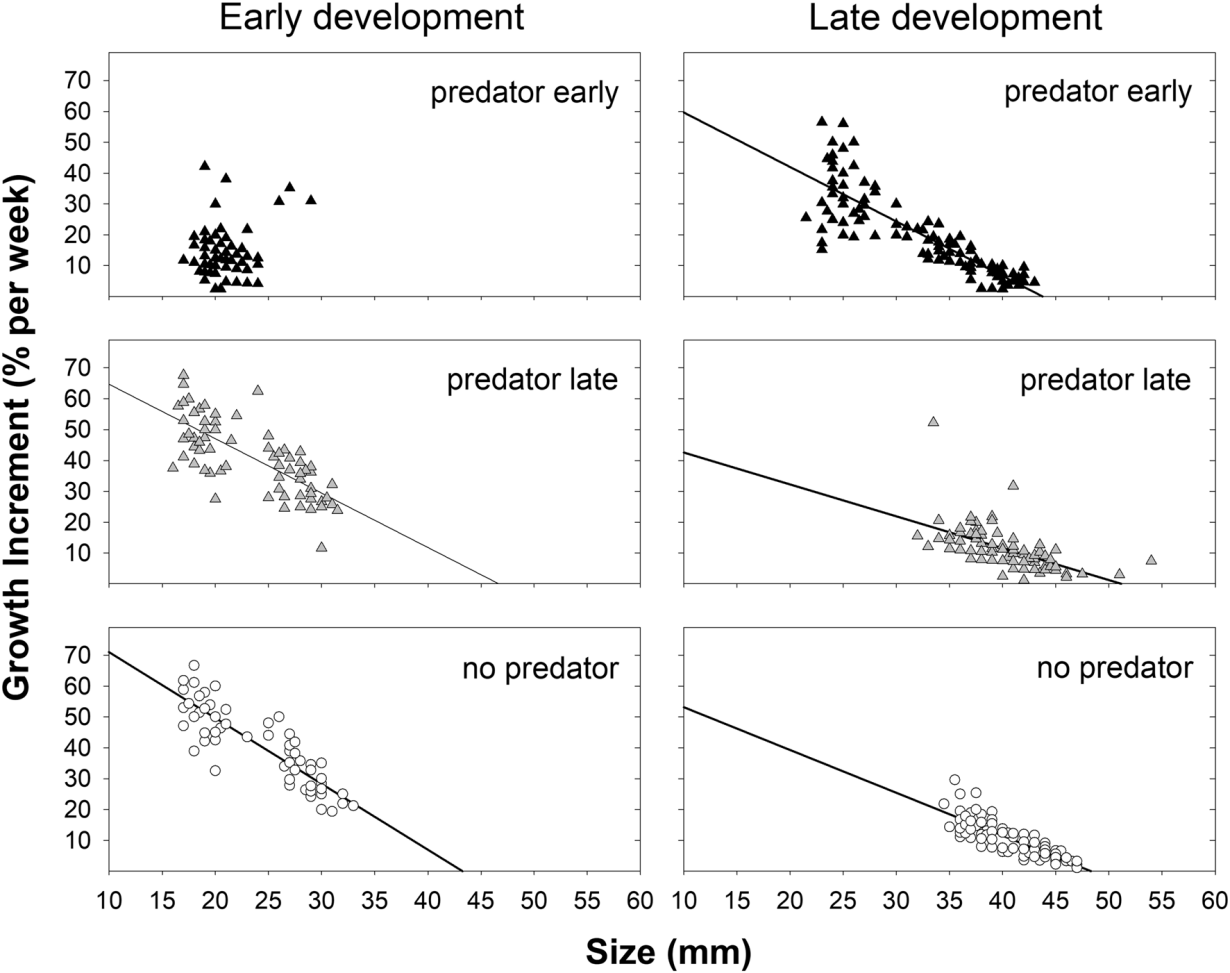
Relationship between growth increment and size throughout two intervals of development. PE treatment—early development: regression NS (p = 0.14), PE treatment—late development: GI (growth increment) = −1.76 size + 77.24, R-square = 0.73; PL treatment—early development: GI = −1.77 size + 82.37, R-square = 0.56, PL treatment—late development: GI = −1.03 size + 52.87, R-square = 0.34; control—early development: GI = −2.13 size + 92.36, R-square = 0.76, control—late development: GI = −1.39 size + 67.00, R-square = 0.71.

The growth increments significantly differed in each time interval (F(2,3) = 306.6, *p* < 0.001, F(2,3) = 31.4, *p* = 0.010, F(2,3) = 9.2, *p* = 0.049, F(2,3) = 78.2, *p* = 0.003, F(2,3) = 20.0, *p* = 0.047). In the PE treatment, the growth increments of the tadpoles differed from those in the PL treatment and the control at all time intervals, while they were similar in the PL treatment and the control (Fig. 3). The presence of predators conspicuously reduced the growth increments of the tadpoles at early development. After the removal of the predator, growth accelerated above the level in the PL treatment and the control (Fig. 3). The growth increments decreased with time and size in the PL treatment and the control (Figs. 3 and 4).

**Figure 5:**
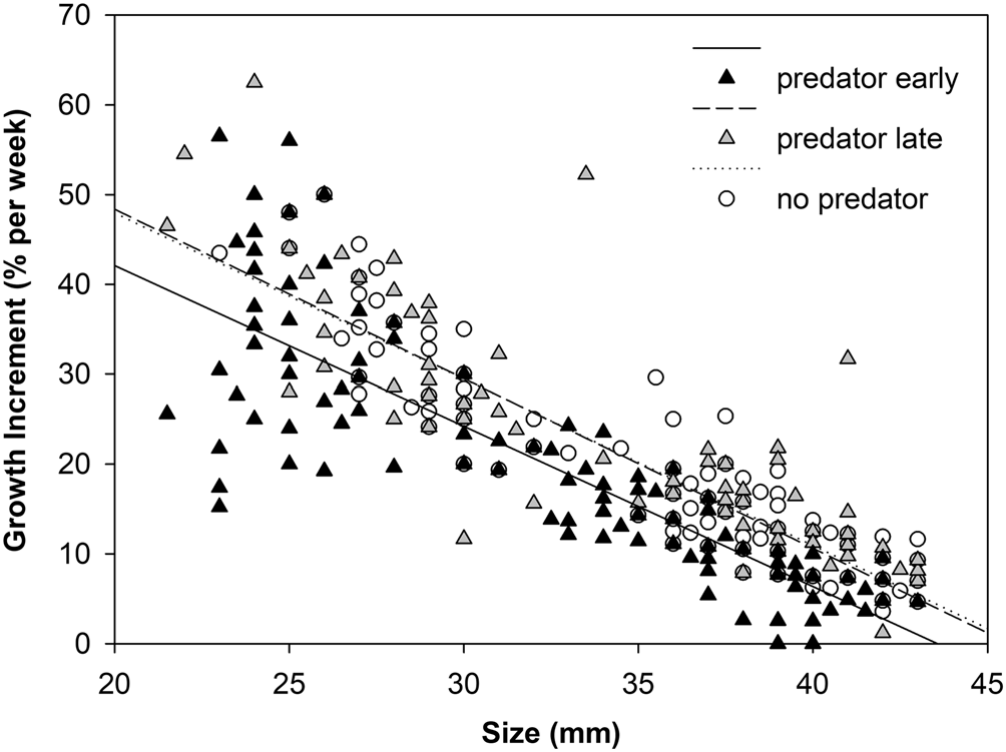
Relationship between growth increment and size for the size categories from 21.5 to 43.0 mm.

However, the growth rate increase after the removal of the predator in the PE treatment was not enough to compensate for the previous growth slowdown (see final size at metamorphosis; Fig. 1). The slopes and intercepts of the individual growth trajectories calculated for the same tadpole size ranges (from 21.5 to 43.0 mm, i.e., the size range available for tadpoles in the PE treatment for intervals 3–5) in all treatments did not differ (F_slopes_ (2,3) = 0.1, *p* = 0.899, F_intercept_ (2,3) = 0.2, *p* = 0.862). Thus, the growth increments of tadpoles that were the same size in the PE and PL treatments and the control were comparable (Fig. 5). Growth compensation after predator removal was therefore not indicated in the PE treatment.

## Discussion

The interaction between predator and prey was investigated in a less frequently studied vertebrate-vertebrate model without the prey being able to attain a “size refuge” (i.e., prey cannot avoid the risk of predation by reaching a threshold size^46^) and with caged predators present only for a defined part of larval prey development. Most previous studies^47–49^ and references there in have addressed the permanent presence of an invertebrate predator and/or fish that occur together throughout the development of tadpoles. In contrast to these studies, we have studied the response of tadpoles to a large gape-unlimited vertebrate predator, which is able to frequently alter its presence in water bodies where tadpoles develop^39^. Moreover, the red-eared slider represents a completely new type of predator in specific places as it is able to colonize even localities in which no other species of turtles live.

Most studies have shown that the continuous presence of a predator has no effect or prolongs the time to metamorphosis and at the same time has no effect or increases the size of the body at metamorphosis^18,47,50–53^. Our results do not match any of these combinations, which could be due to the type of predator used^38^. In accordance with previous results^38^, when a red-eared slider was specifically used as a permanently present predator, common frog tadpoles reached a smaller size at metamorphosis and metamorphosed earlier than without predator. In the present study, we divided the presence of the predator into two intervals, which led to different responses in time to metamorphosis and size/body mass at metamorphosis. The early presence of a predator prolonged the developmental time by 8.5 days (18%), and tadpoles metamorphosed when they were 1.5 mm smaller size (10%) and 0.05 g (17%) lighter than those in the control, while the late presence of a predator did not affect any of these parameters.

Since the presence of a predator reduces the movement activity of tadpoles^37^, and, therefore, food consumption rate, we can compare our results with experiments in which the ontogeny was studied at different food availability^54,55^. The growth trajectory of tadpoles in the PE treatment exactly matches the situation described by Leips and Travis^54^. At an early age, most of the incoming resources are allocated to development, and some resources are allocated to growth; development takes priority. Decreases in food level (corresponding to reduced movement activity^37^ and therefore reduced food intake rate in our PE treatment) at this stage will delay development and prolong the time to metamorphosis. Beyond a certain point of development (late predator presence), the rate of development is fixed, and changes in food level affect only size at metamorphosis^54^. The fact that in our case the PL tadpoles were similar in size and body mass to the control tadpoles may be due to the fact that our switching point lies later than where the developmental rate is fixed, and thus the tadpoles do not have enough time for the difference to become apparent.

Our results for the PL treatment are consistent with the predictions of the Travis model of anuran metamorphosis^43^ (see also Fig. 2b, curves 2 or 3 in Alford and Harris^55^). However, for PE treatment, our results fit neither the Wilbur-Collins^44^ nor the Travis model (see Figs. 1a and 2a, in Alford and Harris^55^). While the Wilbur-Collins model predicts the same size of tadpoles at different times to metamorphosis, the Travis model predicts different sizes at the same time to metamorphosis. We believe that PE tadpoles in our case could grow in agreement with the Wilbur-Collins model. The difference in body size between PE tadpoles and the control, which is not predicted by the model, could then arise because tadpoles are forced to metamorphose within a certain period of time^56,57^, and this time from the release of predator pressure in the PE treatment was not sufficient to allow tadpoles to grow to the same size as tadpoles in the control.

Tadpole vulnerability to predation by both vertebrate and invertebrate predators appears to decrease with increasing tadpole size and age until the onset of metamorphosis^46,58–61^. The lower susceptibility of larger tadpoles to a predator is often explained by the increase in their swimming ability^62,63^ and the decrease in the foraging efficiency of predators on larger tadpoles^58^. The nonsignificant 3% reduction of both size (0.5 mm) and body mass (0.01 g) at metamorphosis in the presence of a red-eared slider in later development with larger tadpoles in comparison to that in the control supports these predictions.

Growth increments were similar in the PL treatment and the control throughout development. In contrast, in the PE treatment, the tadpole increments were conspicuously lower in the presence of a predator and higher in the absence of a predator than in the PL treatment and the control. This result is consistent with the results of Laurila and Kujasalo^64^, where common frog tadpoles developed more slowly early in the experiment in the presence of predators (dragonfly larvae). Yet, their tadpoles metamorphosed later and at larger sizes and were able to catch up with the delay. Other studies have similarly described that when developing individual is placed in better conditions after a period of nutritional deficiency, compensatory growth can occur^65,66^. However, although tadpoles in our study grew faster in the PE treatment after removal of the predator compared to same-age tadpoles in the PL treatment and the control, they were unable to grow to the same size at metamorphosis. The observed higher growth rate in the tadpoles with the early presence of red-eared sliders was the result of the higher growth capacity of smaller tadpoles in this treatment alone, as the growth rates of tadpoles of the same size in the PL treatment and the control were the same. Thus, in our experiment, tadpoles were unable to initiate faster growth above the usual level at a given size after removal of predation pressure but only delayed the normal growth rate until later development.

## Conclusion

The timing of predator–prey interactions has fundamentally different effects at various ontogenetic stages of prey. The predator had the effect on time to metamorphosis, size, and body mass at metamorphosis only if it was present in the early development of tadpoles. In our experiment, the common occurrence of red-eared sliders and common frog tadpoles in early development resulted in a longer time to metamorphosis and a smaller size/body mass of tadpoles than when they occurred together in the later stages of larval development. In amphibians, later metamorphosed individuals may often have a lower survival rate and growth, and a smaller size at metamorphosis is also associated with a smaller size at maturity and reduced reproduction^30,67–69^, but see also^70,71^. Future studies on post-metamorphic stages may provide additional insights into the role of alien predators in common frog antipredator defences.
